# Inflammatory anemia-associated parameters are related to 28-day mortality in patients with sepsis admitted to the ICU: a preliminary observational study

**DOI:** 10.1186/s13613-019-0542-7

**Published:** 2019-06-10

**Authors:** Yi Jiang, Feng-Quan Jiang, Fang Kong, Meng-Meng An, Bei-Bei Jin, Da Cao, Ping Gong

**Affiliations:** 1 Department of Emergency Medicine, General Hospital of Tianjin Medical University, Tianjin City, China; 2 Department of Clinical Laboratory, First Affiliated Hospital of Dalian Medical University, Dalian City, Liaoning Province China; 3 Department of Critical Care Medicine, Huizhou Municipal Central Hospital, Huizhou City, Guangzhou Province China; 4 Department of Emergency Medicine, First Affiliated Hospital of Dalian Medical University, Dalian City, Liaoning Province China

**Keywords:** Iron metabolism, Erythropoietin, Hepcidin, Sepsis, Mortality

## Abstract

**Background:**

Anemia is one of the most common complications of sepsis. Sepsis-related anemia is associated mainly with inflammation. We aimed to observe the changes in the inflammatory anemia-associated parameters of patients with sepsis in the early stage of intensive care unit (ICU) admission and to evaluate their association with 28-day mortality.

**Methods:**

A total of 198 patients with sepsis were divided into survivor (*n* = 110) and non-survivor (*n *= 88) groups on the basis of 28-day survival. Healthy volunteers (*n *= 20) were enrolled as a control group. Plasma levels of iron, ferritin, erythropoietin (EPO), soluble transferrin receptor (sTfR), hepcidin, interleukin-6 (IL-6), hemoglobin and the red blood cell distribution width (RDW) were measured on days 1, 3 and 7 of ICU admission. Clinical data and laboratory findings were collected, and the Sequential Organ Failure Assessment (SOFA) score was calculated.

**Results:**

Patients with sepsis showed significant decreases in hemoglobin, plasma iron and sTfR/log ferritin and significant increases in plasma EPO, sTfR, hepcidin, ferritin and IL-6 on days 1, 3 and 7 of ICU admission compared with healthy volunteers. Hemoglobin was correlated negatively with plasma IL-6 and hepcidin. In patients with sepsis, non-survivors had significantly lower plasma iron, EPO and sTfR/log ferritin, but higher plasma hepcidin, ferritin and IL-6 than survivors on days 1, 3 and 7 of ICU admission. Plasma EPO, hepcidin, ferritin, IL-6, sTfR/log ferritin, the RDW and SOFA score were associated significantly with 28-day mortality but to a varying extent. In particular, in predicting 28-day mortality, plasma hepcidin had an area under the receiver operating curve of 0.808 and 87.3% specificity, which was the highest among the inflammatory anemia-associated parameters tested.

**Conclusions:**

Inflammatory anemia-associated parameters changed significantly in patients with sepsis in the first week of ICU admission. Plasma EPO, hepcidin, ferritin, IL-6, sTfR/log ferritin, the RDW and SOFA score were associated significantly with 28-day mortality. Plasma hepcidin might have a superior predictive value, with high specificity, compared with other inflammatory anemia-associated parameters for 28-day mortality of sepsis patients in the ICU.

**Electronic supplementary material:**

The online version of this article (10.1186/s13613-019-0542-7) contains supplementary material, which is available to authorized users.

## Introduction

Sepsis is a major cause of high mortality in the intensive care unit (ICU) [1]. Anemia is one of the most common complications in patients with sepsis in the ICU [2, 3]. Studies have suggested that sepsis-related anemia can be caused by fluid loading-related hemodilution, iatrogenic blood loss, and decreases in iron supply, erythropoietin (EPO) production and erythrocyte lifespan [2]. However, recently, sepsis-related anemia has been demonstrated to be associated mainly with inflammation (i.e., “anemia of inflammation”) [3–9]. Anemia of inflammation is usually a mild-to-moderately severe anemia (hemoglobin rarely < 8 g/dL) [8, 10]. It develops in the setting of infection, inflammatory disease or malignancy, together with low serum iron despite adequate systemic iron stores, decreased serum transferrin, normal size of erythrocytes and hemoglobin content or mildly decreased size and hemoglobin content of erythrocytes if the inflammatory disease is longstanding [8, 10]. Impaired iron homeostasis and the suppressive effects of pro-inflammatory cytokines on erythropoiesis, together with alterations in the erythrocyte membrane that impair its survival may result, ultimately, in inflammation-associated anemia [8, 11].

Hepcidin is a key regulator of inflammation-associated anemia [12]. Hepcidin reduces the iron level in plasma through (1) direct inhibition of intestinal absorption of iron; (2) promotion of iron storage in macrophages by down-regulating expression of ferroportin in intestinal mucosae and macrophages [12, 13]. Increased interleukin (IL)-6 in patients with sepsis can induce an abruptly increased synthesis of hepcidin [14], causing decreased plasma iron. Plasma iron can be depressed by inflammation markedly (> 50%) and rapidly (24 h) [15].

Also, the interaction between inflammation and iron metabolism may interfere with other inflammatory anemia-associated parameters and complicate iron metabolism in patients with sepsis [7, 8, 12, 16–18]. Typically, plasma ferritin (which stores iron) is reduced in iron deficiency anemia but can increase in the acute phase of sepsis [16, 19]. Anemia usually results in an increased synthesis of EPO in kidneys in minutes to hours [20], but the response to EPO is blunted in patients with sepsis [21]. The soluble transferrin receptor (sTfR), an early and sensitive biomarker for diagnosing iron deficiency [19], is particularly useful for identification of concomitant iron deficiency in patients with inflammation. The sTfR is not affected by inflammation, which is a significant advantage over other biomarkers [22, 23]. Plasma sTfR reflects the degree of iron availability for cells, whereas plasma ferritin reflects iron storage. Hence, the ratio of sTfR to log ferritin (hereafter termed “sTfR/log ferritin”) provides an estimate of the body level of iron and could improve the efficacy of sTfR alone or ferritin alone in the diagnosis of iron deficiency [24].

The inflammatory anemia-associated parameters mentioned above may change with the severity of inflammation in patients with sepsis. The sensitivity and specificity of these parameters can be modified if inflammation and iron deficiency are present concomitantly. This may complicate the diagnosis, evaluation, and treatment of inflammatory anemia-associated anemia in the clinical setting. Hence, a better understanding of changes in the inflammatory anemia-associated parameters of patients with sepsis in the early stage of ICU admission is needed urgently. In addition, although severe anemia is associated with adverse outcomes in critical illness, a lowered plasma iron is part of the natural defense against pathogens [16]. EPO has also been demonstrated to exert protective effects in the kidneys and lungs of mice with sepsis [25], but EPO deficiency contributes to anemia development in patients with sepsis [21]. As a result of these effects, inflammatory anemia-associated parameters have been speculated to be associated with the prognosis of sepsis patients, but relevant studies are lacking.

In this preliminary study, we observed the changes of inflammatory anemia-associated parameters in patients with sepsis in the early stage of ICU admission and evaluated whether these parameters were associated with 28-day mortality.

## Methods

### Ethical approval of the study protocol

The present study was conducted in accordance with the *Declaration of Helsinki* (2013 edition) adopted by the World Medical Association [26]. The study protocol was approved by the Medical Ethics Committee of the First Affiliated Hospital of Dalian Medical University (Dalian, China). Written informed consent was obtained from all patients (or their relatives) upon their initial admission to the hospital and from healthy volunteers.

### Enrolled participants and grouping

This prospective study was conducted in the Emergency ICU of the First Affiliated Hospital of Dalian Medical University. A total of 258 consecutive critically ill patients with sepsis were enrolled from May 1 2016 to November 30 2017. All enrolled patients received full treatment upon ICU admission according to the International Guidelines for Management of Sepsis and Septic Shock [27, 28]. Enrolled patients were subdivided into two groups (survivor and non-survivor) on the basis of 28-day survival. Concurrently, 20 sex- and age-matched healthy adult volunteers were enrolled as a control group.

### Inclusion and exclusion criteria

Patients with sepsis were included in the present study if they met the diagnostic criteria of sepsis [27, 29]. Patients were excluded from this study if they: were < 18 years of age; had chronic liver dysfunctions or chronic kidney disease resulting from different causes, known iron-related diseases (e.g., hemochromatosis), immunologic diseases or malignancies upon hospital admission; had all types of anemia (aplastic, iron deficiency, hemolytic, megaloblastic), trauma before hospital admission or overt blood loss (e.g., gastrointestinal bleeding) upon admission and during the ICU stay; received infusions of blood products during hospitalization (to avoid the undesirable effect of infused blood products on hemoglobin and other anemia-associated parameters); abandoned further treatments.

### Data collection

Clinical data comprising baseline demographic characteristics, medical history, vital signs, the cause of infections and other clinical findings were collected prospectively. The Sequential Organ Failure Assessment (SOFA) score was calculated upon hospital admission on the basis of age, medical history, vital signs, and laboratory results. Samples of venous blood were collected in tubes containing heparin or ethylenediamine tetra-acetic acid in patients with sepsis on days 1, 3 and 7 of ICU admission, or in healthy volunteers upon enrollment. Moreover, blood samples continued to be taken on the ward if patients were discharged alive from the ICU on days 3 or 7. Blood samples were centrifuged (2000×*g*) for 10 min and stored at − 80 °C for further analyses.

Enzyme-linked immunosorbent assay kits were used to measure plasma ferritin (Abcam, Cambridge, MA, USA), EPO (Abcam), sTfR (R&D Systems, Minneapolis, MN, USA), hepcidin (Uscn Life Sciences, Wuhan, China), and IL-6 (USCN Life Sciences). To make the test more specific, the ratio of sTfR (measured in nmol/L) to log ferritin (ng/mL) was calculated. Hemoglobin, mean corpuscular volume, mean corpuscular hemoglobin, mean corpuscular hemoglobin concentration, and red blood cell distribution width (RDW) were measured by an automatic blood cell analyzer (XN-2000; Sysmex, Kobe, Japan).

### Statistical analyses

Data were analyzed using SPSS v22.0 (IBM, Armonk, NY, USA). Data are median and range. We estimated that 57 patients with sepsis in survivor or non-survivor groups (a total of 114) were needed to provide 90% power with a two-sided *α* = 0.05 to detect a 0.5–5.0 difference in three time points (days 1, 3 and 7 of ICU admission) between two groups (survivor and non-survivor) for the change in hepcidin (a main variable in the present study) compared with the baseline value. Pearson Chi-squared or Fisher exact tests were used (as appropriate) to compare demographic variables and the source of sepsis. Variables among time points and groups were compared using a repeated-measure analysis of variance (ANOVA), followed by the Bonferroni test for multiple comparisons. A binary logistical regression analysis for inflammatory anemia-associated parameters of patients with sepsis on day 1 of ICU admission was conducted to determine the factors associated with 28-day survival, and the odds ratios and 95% confidence intervals reported. We used a backward stepwise (conditional) logistic regression method to construct the predictive model. The model was significant in omnibus tests (*P *< 0.001). The accuracy of model prediction was 90.0% in the survival group and 85.2% in the non-survivor group. Receiver operating characteristic (ROC) curves were constructed, and the area under the ROC curve (AUC) were determined. AUCs were compared among the different variables using DeLong’s test. On the basis of the optimal thresholds determined by analyzing ROC curves, prognostic parameters (sensitivity, specificity, positive predictive value [PPV], negative predictive value [NPV], Youden Index, positive likelihood ratio [LR+] and negative likelihood ratio [LR−]) were also calculated. Spearman’s rank correlation coefficient was used to analyze correlations among variables. Differences were considered significant if *P* < 0.05.

## Results

### Baseline characteristics of enrolled participants

**Fig. 1:**
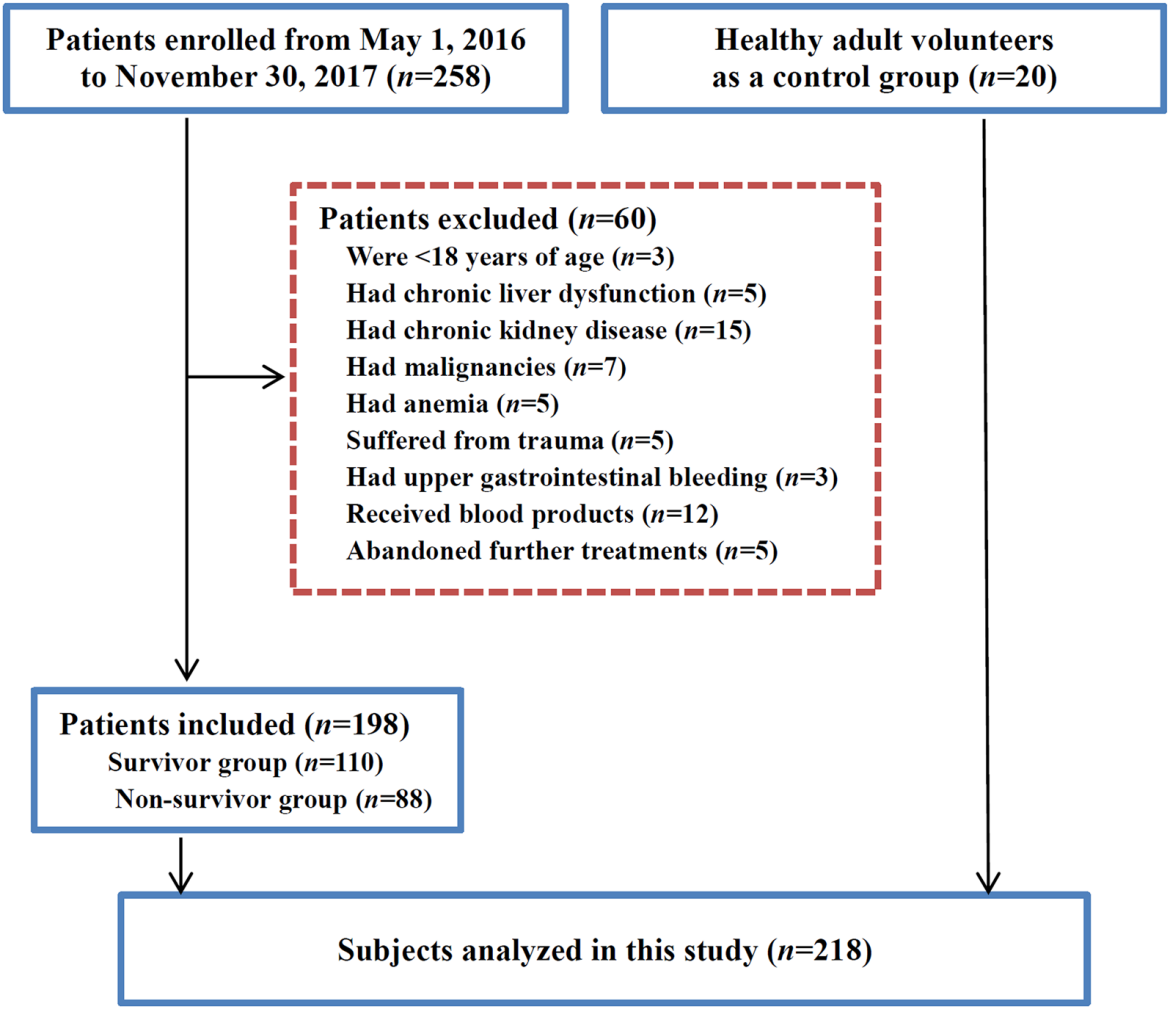
Flowchart of study participants

A total of 198 of the 258 patients with sepsis in this prospective cohort met the inclusion criteria (Fig. 1). Subjects in the survivor group (*n* = 110), non-survivor group (*n* = 88) or control group (*n* = 20) showed no significant difference in age, sex or tobacco use (Table 1). In addition, there were no significant differences in the time from infection onset to ICU admission, length of ICU stay, sources of sepsis, comorbidities, the number of postoperative patients, patients using vasopressors, and hemoglobin between survivor and non-survivor groups (Table 1). However, patients in the non-survivor group had increased use and duration of mechanical ventilation, increased plasma procalcitonin, lactate, creatinine and SOFA score, and decreased PaO_2_/FiO_2_, mean corpuscular volume, mean corpuscular hemoglobin, and mean corpuscular hemoglobin concentration compared with those in the survivor group (all *P* < 0.05).

**Table 1:** Baseline characteristics of the study cohort

	Control (*n *= 20)	Survivor (*n *= 110)	Non-survivor (*n *= 88)	*P* value^a^
Age (years)	65.6 (27–88)	69.9 (28–91)	68.7 (18–91)	0.732
Male [*n* (%)]	12 (60.0%)	74 (67.3%)	52 (59.1%)	0.234
Length of ICU stay (days)	–	15.5 (3.3–76.5)	13.8 (3.5–27.5)	0.535
Sources of sepsis [*n* (%)]				
Lung	–	72 (65.5%)	52 (59.1%)	0.358
Abdomen	–	9 (8.2%)	11 (12.5%)	0.316
Bile	–	22 (20.0%)	16 (18.2%)	0.747
Kidney	–	4 (3.6%)	5 (5.7%)	0.492
Skin/soft tissue	–	3 (2.7%)	4 (4.5%)	0.491
Comorbidities [*n* (%)]				
Hypertension	–	16 (14.5%)	15 (17.0%)	0.630
Diabetes mellitus	–	27 (24.5%)	25 (28.4%)	0.539
Stroke	—	14 (12.7%)	13 (14.8%)	0.677
Acute myocardial infarction	—	2 (1.8%)	1 (1.1%)	0.697
Deep venous thrombosis	—	3 (2.7%)	2 (2.3%)	0.839
Current smoker [*n* (%)]	2 (16.6%)	19 (17.3%)	17 (19.3%)	0.711
Postoperative patients [*n* (%)]	–	12 (10.9%)	10 (11.4%)	0.919
Patients using vasopressors on admission [*n* (%)]	–	6 (5.5%)	4 (4.5%)	0.322
Mechanical ventilation [*n* (%)]	–	41(37.3%)	79 (89.8%)	< 0.01
Ventilation time (hours)	–	151.5 (0–672)	243.2 (0–660)	< 0.01
PaO_2_/FiO_2_	406 (342–463)	244 (121–340)	183 (96–240)	< 0.01
Procalcitonin (ng/mL)	0.02 (0.01–0.04)	3.16 (0.38–56.8)	6.56 (0.87–97.5)	< 0.01
Lactate (mmol/L)	1.0 (0.5–1.7)	2.4 (1.3–3.5)	3.9 (1.8–6.2)	< 0.01
Creatinine (µmol/L)	75 (41–100)	119 (56–188)	138 (65–198)	< 0.01
Hemoglobin (g/L)	135 (130–144)	125 (103–163)	123 (100–161)	0.105
Reticulocytes (×10^9^/L)	87 (76–107)	63 (38–92)	45 (27–68)	< 0.01
Mean corpuscular volume (fL)	90 (85–98)	84 (76–94)	79 (69–89)	< 0.01
Mean corpuscular hemoglobin (pg)	30 (28–33)	29 (24–34)	27 (22–31)	< 0.01
MCHC (g/L)	335 (319–349)	329 (302–352)	306 (290–320)	< 0.01
SOFA score	–	7 (4–13)	10 (6–15)	< 0.01

Values are the median (range)

*ICU* intensive care unit; *NS* not significant; *SOFA* Sequential Organ Failure Assessment; *MCHC* mean corpuscular hemoglobin concentration

^a^Indicates the *P* values for the comparison of survivor and non-survivor groups

### Changes in inflammatory anemia-associated parameters of patients with sepsis in the first week of ICU admission

The hemoglobin concentration decreased gradually over time in patients with sepsis in the first week of ICU admission, indicating anemia on days 3 and 7 (all *P* < 0.05, Table 2). Plasma iron and sTfR/log of sepsis patients in the first week of ICU admission were significantly lower than those of healthy volunteers, together with a slight increase on day 7. However, the plasma hepcidin, EPO and IL-6 of sepsis patients upon ICU admission were significantly higher than those of healthy volunteers and decreased gradually throughout the study, but they remained significantly higher on day 7 than those of healthy volunteers (all *P* < 0.05). Plasma ferritin and sTfR in the first week and the RDW on days 3 and 7 of ICU admission were increased significantly compared with those of healthy volunteers. Also, plasma ferritin was higher on days 3 and 7 than on day 1, but there were no significant differences in plasma sTfR or the RDW among the three time points.

**Table 2:** Changes in inflammatory anemia-associated parameters of patients with sepsis in the first week of ICU admission in sepsis and control groups

	Control (*n *= 20)	Sepsis patients after ICU admission		
		Day 1 (*n *= 198)	Day 3 (*n *= 198)	Day 7 (*n *= 185)
Hemoglobin (g/L)	135 (130–144)	124 (100–163)^a^	103 (78–180)^ab^	95 (81–161)^ab^
RDW (%)	13.1 (12.5–13.5)	13.5 (11.5–19.9)	14.6 (11.9–20.1)^a^	14.5 (11.9–20.4)^a^
EPO (IU/L)	11.2 (9.9–12.6)	73.2 (20.8–428.4)^a^	34.3 (12.1–278.7)^ab^	17.3 (8.1–130.5)^ab^
sTfR (nmol/L)	15.1 (14.0–15.9)	20.3 (13.4–27.9)^a^	20.4 (13.5–28.1)^a^	21.5 (13.4–27.9)^a^
Hepcidin (ng/mL)	21.2 (20.0–22.8)	152.2 (60.2–231.5)^a^	80.6 (24.8–122.4)^ab^	51.1 (20.4–99.7)^ab^
Iron (µmol/L)	19.2 (14.5–20.4)	7.7 (5.3–10.8)^a^	6.7 (5.4–8.8)^a^	8.1 (5.4–11.4)^a^
Ferritin (ng/mL)	30.3 (29.5–31.4)	197.1 (85.2–302.8)^a^	565.6 (340.1–702.4)^ab^	306.4 (99.5–524.6)^ab^
IL-6 (pg/mL)	5.3 (3.0–7.7)	47.9 (15.7–171.9)^a^	33.7 (8.6–153.6)^ab^	33.0 (10.2–107.2)^a^
sTfR/log ferritin	10.2 (9.5–10.8)	8.9 (5.7–12.5)^a^	7.4 (4.8–10.4)^a^	8.8 (5.4–13.2)^a^

Values are the mean ± standard deviation or median (range)

*EPO* erythropoietin; *ICU* intensive care unit; *IL*-*6* interleukin-6; *RDW* red blood cell distribution width; *sTfR* soluble transferrin receptor

^a^*P* < 0.05 *vs.* control

^b^*P* < 0.05 *vs.* day 1 of ICU admission

### Comparison of inflammatory anemia-associated parameters and creatinine levels of patients with sepsis in survivor and non-survivor groups

In the first week of ICU admission, hemoglobin levels decreased gradually over time in survivor or non-survivor patients with sepsis, indicating anemia on days 3 and 7 in both groups (all *P* < 0.05, Table 3), and there were no significant differences between survivor and non-survivor groups at each time point.

**Table 3:** Comparison of inflammatory anemia-associated parameters and creatinine levels of patients with sepsis in survivor and non-survivor groups

	After ICU admission		
	Day 1	Day 3	Day 7
Hemoglobin (g/L)			
Survivor	125 (103–163)	106 (90–180)	95 (81–160)
Non-survivor	123 (100–161)	99 (78–155)	95 (80–112)
RDW (%)			
Survivor	13.3 (11.5–19.0)	14.0 (11.9–19.8)	13.9 (11.9–18.7)
Non-survivor	14.2 (11.8–19.9)	15.4 (12.2–20.1)^a^	15.3 (12.5–20.4)^a^
EPO (IU/L)			
Survivor	93.8 (49.6–428.4)	44.7 (20.0–278.7)^b^	20.7 (10.9–130.5)^b^
Non-survivor	51.3 (20.8–126.2)^a^	23.1 (12.1–81.4)^ab^	11.1 (8.1–29.4)^ab^
sTfR (nmol/L)			
Survivor	20.2 (13.5–27.9)	20.4 (13.5–27.8)	21.7 (13.7–27.9)
Non-survivor	20.7 (13.4–28.0)	20.5 (13.6–28.1)	19.6 (13.4–27.4)
Hepcidin (ng/mL)			
Survivor	133.9 (60.2–185.5)	66.3 (24.8–95.3)^b^	45.7 (20.4–69.3)^b^
Non-survivor	172.2 (110.1–231.5)^a^	99.8 (45.9–122.4)^ab^	65.6 (35.2–99.7)^ab^
Iron (µmol/L)			
Survivor	8.1 (2.3–12.5)	8.6 (2.4–15.4)	10.6 (3.3–19.4)
Non-survivor	7.6 (2.3–15.7)	6.3 (2.9–8.2)^a^	6.6 (3.0–11.4)^a^
Ferritin (ng/mL)			
Survivor	172.5 (85.2–237.1)	555.0 (340.1–615.8)^b^	276.7 (99.5–524.6)^b^
Non-survivor	227.1 (110.7–302.8)^a^	581.9 (423.9–702.4)^ab^	322.5 (218.6–364.6)^a^
IL–6 (pg/mL)			
Survivor	34.2 (15.7–55.5)	23.3 (8.6–45.3)^b^	21.9 (11.7–89.0)^b^
Non-survivor	124.3 (49.3–171.9)^a^	76.0 (29.0–153.6)^ab^	44.9 (10.2–107.2)^ab^
sTfR/log ferritin			
Survivor	9.0 (6.1–12.5)	7.5 (4.9–10.4)	9.3 (5.5–13.2)
Non-survivor	8.8 (5.7–11.9)	6.3 (4.9–10.3)^a^	7.9 (5.4–11.0)^a^
Creatinine (µmol/L)			
Survivor	119 (56–188)	132 (114–201)	124 (67–196)
Non-survivor	138 (65–198)^a^	176 (114–235)^ab^	217 (115–311)^ab^

Values are the mean ± standard deviation or median (range)

*EPO* erythropoietin; *IL*-*6* interleukin-6; *RDW* red blood cell distribution width; *sTfR* soluble transferrin receptor

^a^*P* < 0.05 *vs.* survivor

^b^*P* < 0.05 *vs.* day 1 of ICU admission

Plasma iron and sTfR/log in survivor or non-survivor patients with sepsis in the first week of ICU admission were significantly lower than those of healthy volunteers, but were significantly lower in the non-survivor group than in the survivor group (all *P* < 0.05, Tables 2, 3).

Plasma hepcidin, EPO and IL-6 of survivor or non-survivor patients with sepsis upon ICU admission were significantly higher than those of healthy volunteers and decreased gradually throughout the study. Plasma EPO was decreased significantly, but plasma hepcidin and IL-6 were increased significantly in the non-survivor group compared with those in the survivor group (all *P* < 0.05).

Plasma ferritin and creatinine in survivor or non-survivor patients with sepsis in the first week of ICU admission were significantly higher than those of healthy volunteers, but both were significantly higher in the non-survivor group than in the survivor group (all *P* < 0.05).

The RDW only in non-survivor patients with sepsis was increased significantly on days 3 and 7 of ICU admission compared with that of healthy volunteers, but was significantly higher in the non-survivor group than in the survivor group on days 3 and 7 of ICU admission (all *P* < 0.05).

Plasma sTfR in survivor or non-survivor patients with sepsis was increased significantly in the first week of ICU admission compared with that of healthy volunteers, but there were no significant differences between survivor and non-survivor groups or among each time point.

### Plasma EPO, hepcidin, ferritin, IL-6, sTfR/log ferritin, RDW and SOFA score were associated significantly with 28-day mortality in patients with sepsis

We used 28-day mortality as a dependent variable, and plasma EPO, hepcidin, ferritin, IL-6, sTfR/log ferritin, RDW and SOFA score on day 1 of ICU admission as independent variables. Binary logistic regression analysis showed that plasma EPO, hepcidin, ferritin, IL-6, sTfR/log ferritin, RDW and SOFA score were associated significantly with 28-day mortality in patients with sepsis (Table 4).

**Table 4:** Plasma levels of EPO, hepcidin, ferritin, IL-6, sTfR/log ferritin as well as the RDW and SOFA score on day 1 of ICU admission were independent predictors of 28-day mortality in patients with sepsis

	*β* value	Wald value	*P* value	OR value	95% CI
EPO	− 0.097	7.128	< 0.01	0.908	0.845–0.975
Hepcidin	0.063	8.821	< 0.01	1.065	1.022–1.110
Ferritin	0.010	4.099	< 0.05	1.010	1.000–1.021
IL-6	0.033	6.049	< 0.05	1.033	1.007–1.061
RDW	0.399	4.042	< 0.05	1.490	1.010–2.199
sTfR/log ferritin	− 0.340	5.237	< 0.05	0.712	0.532–0.952
SOFA score	0.435	11.299	< 0.01	1.545	1.199–1.991
Constant	− 12.787	13.431	< 0.01	0.000	

*CI* confidence interval; *EPO* erythropoietin; *ICU* intensive care unit; *IL*-*6* interleukin-6; *OR* odds ratio; *RDW* red blood cell distribution width; *SOFA* Sequential Organ Failure Assessment; *sTfR* soluble transferrin receptor

### Value of inflammatory anemia-associated parameters for predicting 28-day mortality in patients with sepsis

**Fig. 2:**
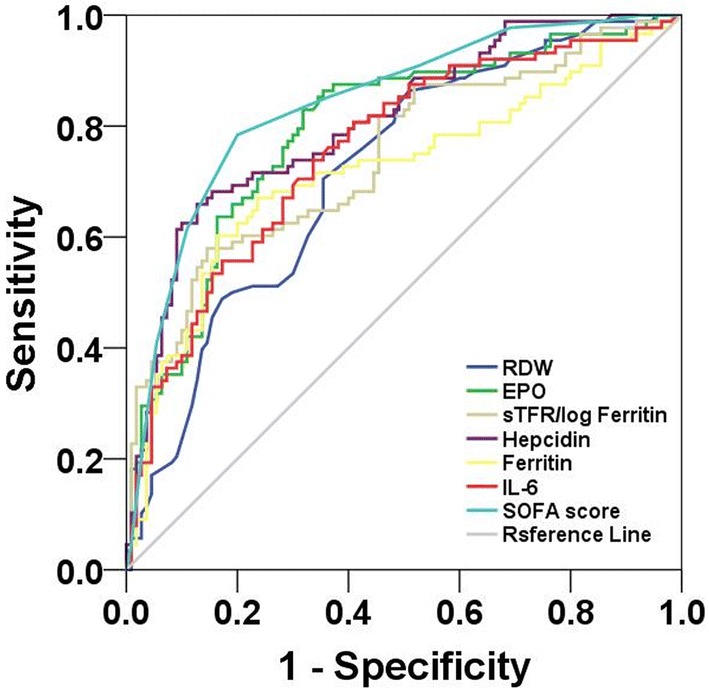
Receiver operating characteristic curves of inflammatory anemia-associated parameters and the SOFA score for predicting 28-day mortality. EPO, erythropoietin; IL-6, interleukin-6; RDW, red blood cell distribution width; SOFA, Sequential Organ Failure Assessment; sTfR, soluble transferrin receptor. The AUCs of plasma EPO (0.794), ferritin (0.720), IL-6 (0.757), sTfR/log ferritin (0.747) and the RDW (0.718) were lower than the SOFA score (0.840) (all *P *< 0.05), but plasma hepcidin (0.808) had an AUC similar to the SOFA score (*P *= 0.28)

Figure 2 shows the ROC curves of plasma EPO, hepcidin, ferritin, IL-6, sTfR/log ferritin, the RDW and SOFA score on day 1 of ICU admission for predicting 28-day mortality. Plasma EPO, hepcidin, ferritin, IL-6, sTfR/log ferritin, the RDW and SOFA score were valuable for predicting 28-day mortality (all *P *< 0.05). Plasma ferritin, IL-6, sTfR/log ferritin and the RDW had lower AUCs than the SOFA score (all *P *< 0.05) but plasma hepcidin had an AUC similar to the SOFA score, which suggested its similarity to the SOFA score for predicting the 28-day mortality of patients with sepsis. Table 5 shows the cutoff value, sensitivity, specificity, PPV, NPV, Youden Index, LR+ , and LR− for plasma EPO, hepcidin, ferritin, IL-6, sTfR/log ferritin, the RDW and SOFA score. Interestingly, hepcidin had the highest specificity.

**Table 5:** Performance of inflammatory anemia-associated parameters and SOFA score on day 1 of ICU admission for predicting 28-day mortality in patients with sepsis

	Cutoff	Sensitivity (%)	Specificity (%)	PPV (%)	NPV (%)	Youden (%)	LR+	LR–
EPO	52.6 IU/L	83.0	68.2	67.6	83.3	51.1	2.61	0.25
Hepcidin	142.6 ng/mL	65.9	87.3	80.6	76.2	53.2	5.19	0.39
Ferritin	207.2 ng/mL	69.3	70.0	64.9	74.0	39.3	2.31	0.44
IL-6	89.1 pg/mL	73.9	66.4	63.7	76.0	40.2	2.20	0.39
RDW	13.7%	86.4	49.1	57.6	81.8	35.5	1.70	0.28
sTfR/log ferritin	8.4 nmol/L	58.0	81.8	71.8	70.9	39.8	3.19	0.51
SOFA score	8.5	78.4	80.0	75.8	82.2	58.4	3.92	0.27

*EPO* erythropoietin; *ICU* intensive care unit; *IL*-*6* interleukin-6; *LR *+ positive likelihood ratio; *LR*– negative likelihood ratio; *NPV* negative predictive value; *PPV* positive predictive value; *RDW* red blood cell distribution width; *SOFA* Sequential Organ Failure Assessment; *sTfR* soluble transferrin receptor

### Correlation among inflammatory anemia-associated parameters and the SOFA score in patients with sepsis

Spearman correlation analysis showed that hemoglobin had a positive correlation with plasma EPO and sTfR/log ferritin (both *P* < 0.05, Additional file 1: Table S6), a negative correlation with plasma IL-6, hepcidin, ferritin and the RDW (all *P* < 0.05), but no correlation with plasma sTfR (*P* = 0.367) in patients with sepsis. Plasma IL-6 had a positive correlation with plasma hepcidin, ferritin and the RDW (all *P* < 0.05) and a negative correlation with plasma EPO and sTfR/log ferritin (both *P* < 0.05). Plasma hepcidin had a positive correlation with plasma ferritin and the RDW (both *P* < 0.05) and a negative correlation with sTfR/log ferritin (*P* < 0.05). Plasma EPO had a positive correlation with sTfR/log ferritin (*P* < 0.05) and a negative correlation with plasma ferritin and the RDW (both *P* < 0.05). The SOFA score had a positive correlation with plasma hepcidin, ferritin, IL-6 and the RDW (all *P *< 0.05) and a negative correlation with plasma EPO and sTfR/log ferritin (both *P *< 0.05).

## Discussion

Patients with sepsis admitted to the ICU seem to develop anemia quickly, which has been evidenced by our study and those of other scholars [3, 4, 7, 17]. Anemia of inflammation and/or iron deficiency anemia often coexists in patients with sepsis in the ICU. This was also evidenced in our study by the decreased hemoglobin together with changes in the parameters associated with anemia of inflammation (increased plasma IL-6, hepcidin, ferritin and decreased plasma iron) and with changes in iron deficiency anemia (increased plasma sTfR and the RDW, and decreased plasma iron). However, it may be difficult to diagnose iron deficiency in the context of anemia of inflammation mainly because there is no accepted “gold standard” for the diagnosis of iron deficiency within the context of inflammation [3, 8]. Moreover, traditional variables, such as sTfR/log ferritin and plasma ferritin, are not accurate for diagnosing iron deficiency in patients with sepsis and even seem contrary to the changes in iron deficiency anemia without inflammation [30], as shown by a decreased sTfR/log ferritin and increased plasma ferritin in our study. However, in our study, iron deficiency was observed in patients with sepsis from day 3 of ICU admission if diagnosed with an increased sTfR and decreased plasma iron as well as hepcidin < 129.5 ng/mL [31].

Erythropoiesis has been documented to be affected severely by inflammation [20, 21, 32], and inflammation-associated abnormalities in erythropoiesis contribute to early-onset anemia, which has been found in patients with septic shock [32]. Erythropoiesis is regulated strictly by EPO [33]. EPO induces the production of erythroferrone (a glycoprotein hormone secreted by erythroblasts) and suppresses hepcidin and, thus, facilitates iron delivery during stress erythropoiesis [34]. The erythropoietic effect of EPO can be blunted by tumor necrosis factor-α as well as through down-regulation of EPO receptors [32, 35]. Hence, inflammation can itself inhibit the erythropoietic effects of EPO. In addition, plasma EPO is usually increased in response to anemia and hypoxemia. However, plasma EPO was not as high as expected and fell quickly, but was increased compared with that in healthy volunteers in the present study. This may have been because of a combination of impaired renal function (which decreases EPO production) and inhibition of EPO production by pro-inflammatory cytokines in critically ill patients [21, 36]. We speculated that enhancement of EPO production by anemia and hypoxemia might overwhelm inhibition of EPO production by inflammation in patients with sepsis. Moreover, other confounding factors may have interfered with the correlation of EPO and anemia, which would have contributed to the relatively weak correlations observed in our study, and so had relatively weak correlations between other parameters.

We also found non-surviving sepsis patients to have lower plasma EPO than that in surviving patients, but there were no differences in hemoglobin between surviving and non-surviving sepsis patients, a result that is consistent with data from other studies [21, 33]. This phenomenon may have been due (at least in part) to more severe inflammation that resulted in dominant inhibition of EPO production and worse renal function that decreased EPO production directly in non-surviving sepsis patients than surviving sepsis patients. In addition, the complicated association of EPO with inflammation and anemia described above could partly explain why plasma EPO was associated significantly with 28-day mortality.

Iron metabolism is also affected severely by inflammation [2, 6, 9, 12, 14, 16–18]. Imbalances in iron metabolism occur quickly after ICU admission along with the inflammatory process, as indicated by the decreased plasma iron and sTfR/log ferritin and increased plasma sTfR, hepcidin, ferritin and IL-6 in the first week of ICU admission shown in our study. However, the effect of low plasma iron on patients with sepsis seems inconsistent. On the one hand, a low iron level may have a protective effect [4]. Iron is an essential micronutrient for nearly all microorganisms and, therefore, a low iron level limits the harm of microorganisms to the host [37]; free iron is also toxic because it results in the generation of reactive oxygen species, lipid peroxidation, and endothelial damage; moreover, iron content in macrophages regulates their cytokine production [16]. On the other hand, a too low level of iron may lead to low iron availability and iron deficiency anemia and, thus, be associated with adverse outcomes of patients with sepsis [30]. This might also have been evidenced by lower plasma iron in non-surviving patients with sepsis than surviving patients with sepsis in our study. Hence, the inconsistent effect of a low iron level described above may explain the inconsistent results (harmful, effective or ineffective) in many studies on iron supplementation (oral or intravenous) in critically ill patients [8, 38]. Also, we must consider the complexity of mechanisms of sepsis-related anemia and interaction of relevant parameters: the non-availability of iron for erythropoiesis despite high iron reserves in tissue; a blunted erythropoietic effect of EPO by pro-inflammatory cytokines; suppression of hepcidin by erythroferrone (thus facilitating iron delivery during stress erythropoiesis); erythroferrone production induced by EPO; regulated systemic uptake and recycling of iron by hepcidin through inhibition of the hepcidin–ferroportin interaction [2, 8, 9, 17, 34, 35, 38, 39]. Hence, some therapeutic strategies, such as iron supplementation or hepcidin antagonism, may fail to improve this anemia and cause serious adverse effects if applied alone.

In addition, we found no difference in plasma sTfR between surviving and non-surviving sepsis patients. Blunted EPO production and suppressed erythropoiesis by cytokines may be reasons for an absence of increase in sTfR in non-surviving sepsis patients despite lower plasma iron and higher RDW in comparison with those of surviving patients [24].

In addition to IL-6, the RDW and SOFA score (which have been used previously as prognostic variables [40–44]), iron metabolism-associated parameters such as plasma hepcidin, ferritin and sTfR/log ferritin were associated significantly with 28-day mortality and were valuable for predicting 28-day mortality in the present study. However, the odds ratios for hepcidin, ferritin and IL-6 were low. We speculated that the low odds ratios might have been associated with complicated mechanisms of inflammation-associated anemia, and that several confounding factors could interfere with these associations. Also, only plasma hepcidin had a superior predictive value, with the highest specificity, compared with other inflammatory anemia-associated parameters. The superior value of hepcidin in predicting 28-day mortality may be associated with its causal role in sepsis-related anemia and with its role as a potential acute‑phase biomarker related to illness severity in inflammation and sepsis [6, 9, 39, 45–47]. However, this result is not consistent with a study by Tacke and colleagues [4]. They showed that non-surviving critically ill patients tended to have a non-significantly higher hepcidin than surviving critically ill patients during 3-year follow-up [4]. The discrepancy may have been due to different selection criteria for patients. Tacke and colleagues enrolled sepsis and non-sepsis ICU patients, whereas we enrolled only sepsis patients admitted to the ICU. However, non-sepsis ICU patients might be confounding factors for predicting 28-day mortality due to their lower hepcidin level and milder inflammatory response than sepsis patients in the ICU.

In addition, low plasma hepcidin upon ICU discharge was found to be an independent predictor of one-year mortality in critically ill patients in a study by Lasocki and colleagues [30], which seems inconsistent with our results. This inconsistency arose from the different time points and different physical status of patients. Plasma hepcidin in the study of Lasocki and co-workers was measured upon ICU discharge, when patients might have mild inflammation and severe iron deficiency, thereby indicating the dominant effect of iron deficiency upon hepcidin and the prognosis. In contrast, plasma hepcidin in our study was measured upon ICU admission, when patients might have severe inflammation with mild or no iron deficiency, thereby indicating the dominant effect of inflammation on hepcidin and the prognosis. Hence, interpretation of the prognostic value of hepcidin should be conducted carefully, and the status of critically ill patients should also be considered.

The present study had six main limitations. First, the study cohort was relatively small because of the short study period and strict exclusion criteria, but we will enroll more patients to increase the statistical power in future studies. Second, patients diagnosed with iron deficiency anemia in the outpatient setting were not enrolled as a control population. Third, we did not assess differences in subgroups on the basis of sex, but the sex distribution in the two groups was even. Fourth, hypoxemia with a partial pressure of oxygen < 75 mmHg is a potent stimulus for EPO production [21], but we failed to undertake subgroup analyses of a hypoxemia group. Fifth, we failed to measure zinc protoporphyrin in addition to RDW as markers of iron-deficient erythropoiesis during the study. Finally, we excluded patients who received infusions of blood products during hospitalization, so our findings may not be generalizable to patients who receive a blood transfusion (which represents substantial proportion of ICU patients).

## Conclusions

Inflammatory anemia-associated parameters changed significantly in patients with sepsis in the first week of ICU admission. Plasma EPO, hepcidin, ferritin, IL-6, sTfR/log ferritin, the RDW and SOFA score were associated significantly with 28-day mortality. In particular, plasma hepcidin might have a superior predictive value, with high specificity, compared with other inflammatory anemia-associated parameters for 28-day mortality of sepsis patients in the ICU.

## Additional file

**Additional file 1: Table** **S6.** Correlation among inflammatory anemia-associated parameters and the SOFA score.

## Data Availability

The datasets analyzed during the current study are available from the corresponding author on reasonable request.
