# Supplementary material for: Inflammatory anemia-associated parameters are related to 28-day mortality in patients with sepsis admitted to the ICU: a preliminary observational study
Source: Ann Intensive Care. 2019 Jun 10;9:67. doi: 10.1186/s13613-019-0542-7 (PMC6557959; doi:10.1186/s13613-019-0542-7)
Supplement: Supplementary file 1 — Additional file 1: Table S6. Correlation among inflammatory anemia-associated parameters and the SOFA score. [file 13613_2019_542_MOESM1_ESM.docx]

Additional file 1: Table S6. Correlation among inflammatory anemia-associated parameters and the SOFA score

|  | **Hemoglobin** | | **Hepcidin** | | | **Ferritin** | | | **IL-6** | | | **EPO** | | | **RDW** | | | **sTfR/logFerritin** | | | **SOFA score** | | | |
| --- | --- | --- | --- | --- | --- | --- | --- | --- | --- | --- | --- | --- | --- | --- | --- | --- | --- | --- | --- | --- | --- | --- | --- | --- |
|  | ***r*** | ***P*** | | ***r*** | ***P*** | | ***r*** | ***P*** | | ***r*** | ***P*** | | ***r*** | ***P*** | | ***r*** | ***P*** | | ***r*** | ***P*** | | ***r*** | ***P*** |  |
| **Hemoglobin** | ‒ | ‒ | | -0.296 | < 0.01 | | -0.420 | < 0.01 | | -0.375 | < 0.01 | | 0.463 | < 0.01 | | -0.260 | < 0.01 | | 0.208 | < 0.01 | | -0.052 | 0.465 |  |
| **Hepcidin** | -0.296 | < 0.01 | | - | - | | 0.520 | < 0.01 | | 0.380 | < 0.01 | | -0.556 | < 0.01 | | 0.430 | < 0.01 | | -0.202 | < 0.01 | | 0.288 | < 0.01 |  |
| **Ferritin** | -0.420 | < 0.01 | | 0.550 | < 0.01 | | - | - | | 0.340 | < 0.01 | | -0.567 | < 0.01 | | 0.530 | < 0.01 | | -0.294 | < 0.01 | | 0.259 | < 0.01 |  |
| **IL-6** | -0.375 | < 0.01 | | 0.380 | < 0.01 | | 0.340 | < 0.01 | | - | - | | -0.300 | < 0.01 | | 0.295 | < 0.01 | | -0.163 | < 0.05 | | 0.245 | < 0.01 |  |
| **EPO** | 0.463 | < 0.01 | | -0.556 | < 0.01 | | -0.567 | < 0.01 | | -0.300 | < 0.01 | | - | - | | -0.505 | < 0.01 | | 0.312 | < 0.01 | | -0.261 | < 0.01 |  |
| **RDW** | -0.260 | < 0.01 | | 0.430 | < 0.01 | | 0.530 | < 0.01 | | 0.295 | < 0.01 | | -0.505 | < 0.01 | | - | - | | -0.223 | < 0.01 | | 0.324 | < 0.01 |  |
| **sTfR/log ferritin** | 0.208 | < 0.01 | | -0.202 | < 0.01 | | -0.294 | < 0.01 | | -0.163 | < 0.05 | | 0.312 | < 0.01 | | -0.223 | < 0.01 | | - | - | | -0.129 | < 0.05 |  |
| **SOFA score** | -0.052 | 0.465 | | 0.288 | < 0.01 | | 0.259 | < 0.01 | | 0.245 | < 0.01 | | -0.261 | < 0.01 | | 0.324 | < 0.01 | | -0.129 | < 0.05 | | ‒ | ‒ |  |

EPO, erythropoietin; IL-6, interleukin-6; RDW, red blood cell distribution width; SOFA, Sequential Organ Failure Assessment; sTfR, soluble transferrin receptor.
